# The Impact of Follow-Up on Etiological Classification of Pediatric Vertigo

**DOI:** 10.3390/children13030376

**Published:** 2026-03-06

**Authors:** Nina Božanić Urbančič, Dejan Mladenov, Saba Battelino

**Affiliations:** 1 Department of Otorhinolaryngology and Cervicofacial Surgery, University Medical Centre Ljubljana, Zaloška cesta 2, 1000 Ljubljana, Slovenia; dejan.mladenov@kclj.si (D.M.); saba.battelino@kclj.si (S.B.); 2 Faculty of Medicine, University of Ljubljana, Vrazov trg 2, 1000 Ljubljana, Slovenia

**Keywords:** pediatric vertigo, dizziness, follow-up, etiology, vestibular disorders, migraine

## Abstract

**Highlights:**

**What are the main findings?**

- Extended longitudinal follow-up dramatically reduced the proportion of pediatric vertigo with initially unknown etiology, from 43% at first evaluation to 10% after diagnostic reassessment.
- Many cases initially classified as unexplained vertigo evolved into identifiable clinical entities, most commonly migraine-related vertigo, as well as central, hemodynamic, psychogenic, and peripheral vestibular disorders.

**What are the implications of the main findings?**

- In pediatric vertigo, follow-up should be regarded as an active diagnostic tool, not merely observation, because clinical phenotypes often emerge over time.
- Early diagnostic uncertainty is common in children with vertigo and should prompt structured longitudinal evaluation rather than premature diagnostic closure.

**Abstract:**

**Background:** Vertigo and dizziness in children represent diagnostically challenging conditions with heterogeneous etiologies. At initial presentation, a substantial proportion of pediatric patients remain without a definitive etiological diagnosis. Evidence on the impact of longitudinal follow-up on etiological classification in pediatric vertigo is limited. **Methods:** This observational cohort study uses prospectively collected clinical data. Children aged 1–17 years who presented to a tertiary ENT clinic with vertigo and/or dizziness between 2015 and 2020 were systematically enrolled and followed. The present study represents a retrospective revision of a previously published cohort of 257 children. In 2025, extended follow-up data were reviewed to reassess etiological classification using the same diagnostic categories as in the original analysis. Descriptive statistics were applied to compare etiological distributions at initial evaluation and after follow-up revision. **Results:** After data revision, the proportion of children with unclassified etiology decreased from 44% to 10%. Central etiologies accounted for 35% of cases, peripheral vestibular disorders for 18%, hemodynamic causes for 16%, psychogenic etiologies for 10%, and other specific causes for 7%. Follow-up duration ranged from 0 to 132 months (mean 17.6 months; median 4.5 months). Diagnostic investigations were frequently performed; however, the etiological yield of certain tests, particularly cranial computed tomography, was low. **Conclusions:** Extended follow-up significantly improves etiological classification in children with vertigo and dizziness, demonstrating that diagnostic uncertainty at initial presentation often reflects evolving clinical phenotypes rather than the absence of an underlying disorder. A longitudinal, clinically guided, and multidisciplinary approach is essential to enhance diagnostic accuracy and optimize the use of diagnostic investigations in pediatric vertigo.

## 1. Introduction

Vertigo and dizziness in childhood represent complex clinical entities with diverse etiologies, spanning central vestibular disorders, peripheral vestibulopathies, systemic factors, and psychogenic components. Although pediatric vertigo is less commonly encountered than in adults, its impact on quality of life, cognitive function, and psychosocial development can be substantial if not appropriately diagnosed and managed [1,2].

Epidemiological studies indicate that migraine-related syndromes—including vestibular migraine and its pediatric variants such as benign paroxysmal vertigo of childhood (BPVC), now often termed recurrent vertigo of childhood—are among the most frequent causes of vertigo in children, followed by peripheral vestibular disorders and less common conditions such as vestibular neuritis and psychogenic vertigo [1,3,4].

Benign paroxysmal vertigo of childhood, a condition frequently linked to migraine spectrum disorders, is characterized by episodic vertiginous spells in the absence of a persistent vestibular lesion and is among the most common identifiable causes of vertigo in young children [5,6]. The International Classification of Headache Disorders recognizes these migraine-related variants, reflecting their central neurological origin and often overlapping clinical features with migraine itself [2].

Despite advances in diagnostic techniques and increasing awareness among clinicians, a substantial proportion of pediatric patients still present with vertigo or dizziness that cannot be definitively classified at initial presentation. Large retrospective series have reported that approximately 20% of cases have an undetermined etiology after the first clinical evaluation [4]. This diagnostic uncertainty poses challenges for prognostication, therapeutic decision-making, and patient counseling.

Longitudinal follow-up studies in pediatric vestibular populations are comparatively scarce, yet evidence suggests that etiological classifications may evolve over time, particularly as clinical features mature, new symptoms emerge, or additional diagnostic information becomes available [7]. Such temporal evolution is especially notable for conditions like recurrent vertigo of childhood and vestibular migraine, where initial presentations may not fully meet diagnostic criteria but subsequently develop into more defined syndromes.

In our previously reported cohort from a tertiary referral center, a significant proportion of children presenting with vertigo and dizziness were classified as having an unexplained etiology following multidisciplinary assessment [8]. In most cases, follow-up evaluation was deemed clinically necessary and therefore scheduled at the initial visit. After systematic revision of the cohort in 2025, incorporating extended clinical follow-up and updated diagnostic information, we observed that many children initially labeled as unclassified had subsequently received specific diagnoses. These findings underscore the dynamic nature of pediatric vertigo and highlight the diagnostic value of longitudinal evaluation.

Therefore, the present study aims to re-evaluate the etiological distribution in our pediatric vertigo cohort after extended follow-up, focusing on changes in classification and the implications for clinical practice. By comparing initial and follow-up etiological assignments, we seek to quantify how longitudinal data influence diagnostic accuracy and to inform recommendations for follow-up strategies in pediatric vestibular disorders.

## 2. Materials and Methods

### 2.1. Study Design and Population

This study is an observational cohort study based on prospectively collected clinical data. Children presenting with vertigo and dizziness have been systematically enrolled and followed since 2015. The present analysis represents a retrospective review and revision of etiological classification using extended follow-up data.

The original cohort included consecutive children and adolescents aged 1–17 years who presented with vertigo and/or dizziness and were referred for multidisciplinary evaluation between 2015 and 2020.

In 2025, a systematic collection and revision of the clinical data was conducted, incorporating additional follow-up information collected after the initial publication. The aim of the revision was to reassess etiological classification in light of newly available diagnostic information that emerged during long-term follow-up.

No new patients were added to the cohort. The study population, therefore, corresponds exactly to the cohort described in the original publication.

### 2.2. Diagnostic Workup and Follow-Up

All children underwent a comprehensive diagnostic evaluation at initial presentation, as described previously, including detailed medical history, otoneurological examination, age-appropriate audiological and vestibular testing, and referral to relevant pediatric subspecialists (pediatric neurology, cardiology, psychology/psychiatry, ophthalmology, infectious disease, and radiology) based on the clinical presentation. Follow-up was scheduled in children with: persistent or recurrent symptoms, undefined etiology, suspected migraine phenotype, or abnormal neurological or vestibular findings. No strictly standardized intervals were applied; follow-up was clinically tailored.

Follow-up data of regular check-ups were obtained from medical records documenting subsequent outpatient visits, hospital admissions, specialist consultations, and diagnostic investigations performed after the initial evaluation. Particular attention was paid to children who were originally classified as having vertigo and dizziness of unclassified etiology.

Vestibular assessment was adapted to the child’s age and level of cooperation and included bedside examination and, when feasible, instrumental testing such as video head impulse testing (vHIT-Interacustics, Middelfart, Denmark), caloric testing (Variotherm plus ATMOS MedizinTechnik GmbH and Co. KG Ludwig-Kegel, Lenzkirch, Germany), and vestibular evoked myogenic potentials (cVEMP; Eclipse, Interacustics A/S, 6610 Assens, Denmark). In younger children, completion of instrumental testing was occasionally limited by cooperation and developmental factors.

During follow-up, etiological reclassification was performed when sufficient clinical, diagnostic, or specialist-confirmed information became available to support a specific diagnosis. Diagnoses established during follow-up were based on accepted clinical criteria relevant at the time of reassessment (e.g., migraine-related disorders, autonomic/hemodynamic disorders, central neurological conditions, psychological disorders). Migraine-related vertigo was diagnosed according to Bárány Society and ICHD-3 criteria. BPPV was diagnosed based on positional nystagmus consistent with canal involvement. Vestibular neuronitis was defined by acute, prolonged vertigo, compatible vestibular findings, and the absence of central signs.

### 2.3. Etiological Classification

Etiological categories were identical to those used in the original study to allow direct comparison between initial and revised classifications. These included: central causes, peripheral vestibular causes, hemodynamic causes, psychological causes, other identified causes, and unclassified etiology.

Reclassification was conducted by reviewing follow-up documentation and specialist reports. A diagnosis was considered revised only when supported by documented clinical assessment and/or diagnostic testing performed during follow-up. Psychogenic etiologies were diagnosed after exclusion of organic causes and, when indicated, in collaboration with pediatric neurology or psychology services. No diagnosis was made based solely on the absence of findings.

Serological testing for Borrelia burgdorferi was performed only when clinically indicated (e.g., compatible neurological symptoms, tick exposure, or endemic area).

### 2.4. Data Analysis

Descriptive statistics were used to summarize demographic characteristics and etiological distributions at initial evaluation and after follow-up revision. Categorical variables were expressed as frequencies and percentages. Continuous variables were summarized using means and standard deviations or medians and ranges, as appropriate.

Comparisons between initial and revised etiological classifications were performed to quantify changes in diagnostic distribution, with particular focus on the proportion of children initially classified as having an unclassified etiology who subsequently received a definitive diagnosis.

Statistical analysis was done using SPSS V20.0 (IBM, Armonk, NY, USA) and Microsoft Excel 2019 (Microsoft, Redmond, WA, USA). *p* < 0.05 was considered statistically significant.

## 3. Results

### 3.1. Study Population

A total of 257 children were included in the revised analysis. The cohort consisted of 42% males and 58% females, identical to the previously published population. The age range and demographic characteristics remained unchanged, as no new patients were added.

### 3.2. Overall Etiological Distribution After Revision (2025)

After extended clinical follow-up and evaluation of collected data, the etiology of vertigo and dizziness was reclassified in the majority of children. Most reclassifications occurred within the first two follow-up visits.

**Figure 1:**
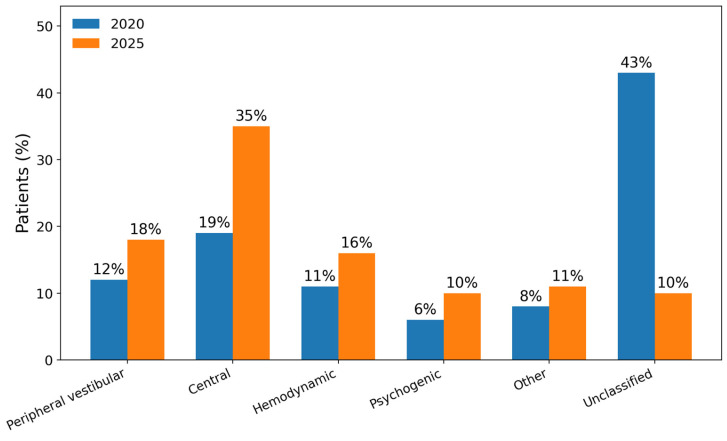
Comparison of etiological groups at initial evaluation (2020) and after follow-up revision (2025). Extended follow-up substantially reduced the proportion of unclassified cases and increased the identification of central, hemodynamic, psychogenic, and peripheral vestibular etiologies.

In total, 26 children (10%) remained classified as having unclassified etiology (Figure 1).

**Figure 2:**
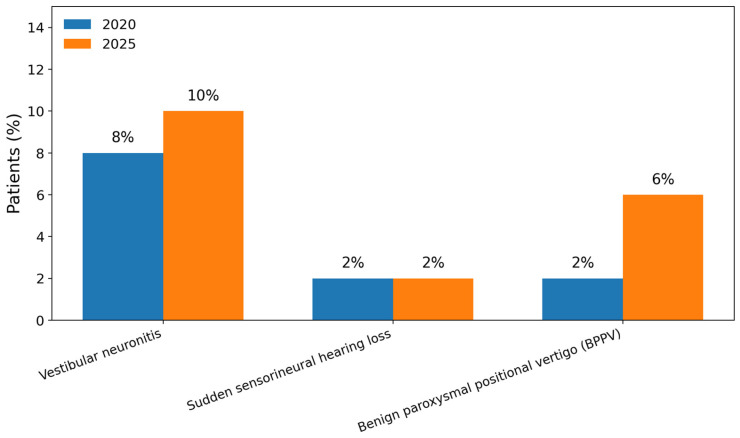
Etiological distribution of peripheral causes of pediatric vertigo and dizziness before (2020) and after data revision in 2025.

**Figure 3:**
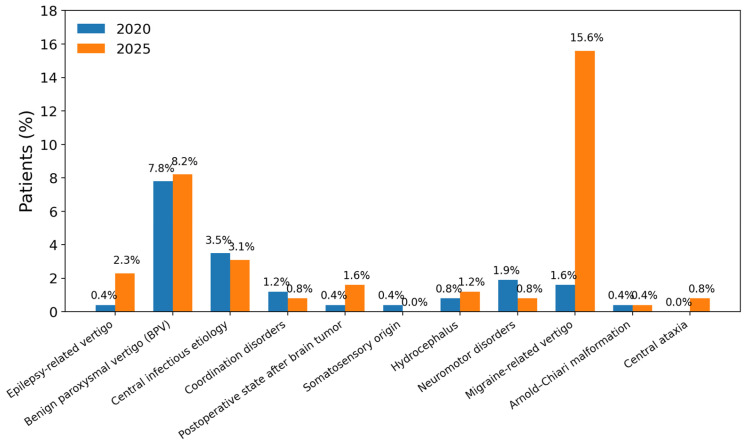
Distribution of central etiologies by diagnosis at initial evaluation (2020) and after follow-up revision (2025). Migraine-related vertigo emerged as the predominant central cause following extended follow-up, highlighting the diagnostic impact of longitudinal assessment.

Figure 2 and Figure 3 illustrate the distribution of peripheral and central etiologies after data revision. Among peripheral causes, benign paroxysmal positional vertigo predominated, while migraine-related vertigo represented the most frequent central diagnosis.

### 3.3. The Follow-Up Assessment

Follow-up data were available for 256 children. The duration of follow-up ranged from 0 to 132 months, with a mean follow-up time of 17.6 months, a median of 4.5 months, and an interquartile range (IQR) of 0–24 months. A follow-up duration of “0 months” indicates cases evaluated at a single clinical encounter with no subsequent scheduled reassessment.

Of the total cohort (N = 257), 52 children (20%) were not scheduled for follow-up, while 29 children (11%) did not attend the planned follow-up visit, resulting in 32% of children without a completed follow-up examination. Table 1 shows the extended multidisciplinary assessment during the follow-up. Neurological examination was performed in 190 children (74% of the total cohort). Of these, 164 children (86%) had normal neurological status, while 26 (14%) had neurological abnormalities. EEG examination was performed in 94 children (36%). Normal EEG findings were observed in 85 children (90%), while 9 children (10%) showed pathological EEG findings. Tilt-table testing was performed in 13 children (5% of the total cohort). Normal findings were observed in 5 children (39%), while 8 children (62%) showed pathological results. The majority of cases were consistent with orthostatic intolerance and reflex-mediated mechanisms; formal autonomic testing was not systematically performed in all patients.

Cranial CT imaging was performed in 160 children (62% of the total cohort). Normal CT findings were observed in 158 children (99%), while 2 children (1%) showed pathological findings.

Brain MRI was performed in 146 children (57% of the total cohort). Normal MRI findings were observed in 121 children (83%), while 25 (17%) showed abnormal findings.

Serological testing for Borrelia burgdorferi was performed in 78 children (30% of the total cohort). Negative results were observed in 71 children (91%), while 7 children (9%) tested positive.

Ophthalmological examination or follow-up was performed in 84 children (33% of the total cohort). Of these, 13 children (15%) showed pathological ophthalmological findings.

## 4. Discussion

### 4.1. Longitudinal Reclassification of Etiology: Comparison Between 2021 and 2025

In contrast to our 2021 report, in which the etiology of vertigo and dizziness remained unclassified in 44% (112/257) of children [8], the 2025 data revision demonstrated a marked reduction in unclassified cases to 10% (26/257). This finding underscores that pediatric vertigo is frequently an evolving clinical entity, with diagnostic clarity often emerging only with longitudinal follow-up. Similar trends have been reported in recent pediatric cohorts, where extended observation and standardized diagnostic frameworks substantially reduced the proportion of unexplained cases [9,10]. Table 2. Demonstrates the percentage of unexplained pediatric vertigo and dizziness cases in different studies.

Recent single-center cohort studies have described the etiological spectrum of pediatric vertigo based predominantly on findings at initial presentation, without longitudinal reclassification; while these studies provide valuable cross-sectional insights, they do not capture the dynamic evolution of diagnoses over time, which may contribute to persistently high rates of unclassified cases [14]. In contrast, our longitudinal revision demonstrates that extended follow-up substantially improves etiological classification, supporting follow-up as an active diagnostic tool rather than a passive observational period.

While unclassified cases decreased, diagnoses within central, hemodynamic, psychogenic, and peripheral categories increased. Specifically, after the 2025 revision, central etiologies accounted for 35% (91/257) of cases, peripheral vestibular disorders for 18% (45/257), hemodynamic causes for 16% (41/257), psychogenic etiologies for 10% (26/257), and other specific etiologies for 7% (19/257), with only 10% remaining unclassified. On the other hand, considerable variability in epidemiological data across published studies likely reflects differences in study design, referral patterns, the professional backgrounds of investigators (ENT specialists, neurologists, pediatricians), diagnostic protocols, and the timing of evaluation. Furthermore, heterogeneity in bedside versus instrumental investigations and emergency versus outpatient assessment may substantially influence reported etiological distributions. These methodological differences partly explain discrepancies in the proportion of unclassified cases reported in the literature.

In comparison, the 2021 analysis reported substantially lower proportions for several of these categories, suggesting that initial diagnostic uncertainty often reflects incomplete phenotypic expression rather than the absence of an underlying disorder. This longitudinal shift mirrors findings from recent studies indicating that standardized classification and follow-up significantly improve etiological resolution in pediatric dizziness [15]. These findings further support the concept that pediatric vertigo represents a distinct clinical entity, requiring age-specific diagnostic pathways and longitudinal follow-up strategies, rather than extrapolation from adult data. Moreover, our findings support the concept of a “diagnostic waiting period” in pediatric vestibular medicine, in which longitudinal follow-up serves as an active diagnostic tool rather than passive observation. Symptom evolution over time allows more accurate etiological classification, particularly in migraine-related and developmentally evolving conditions.

### 4.2. The Follow-Up

The duration and structure of follow-up in our cohort reflect a clinically tailored approach rather than uniform longitudinal surveillance. Children who were not scheduled for follow-up represented cases in which comprehensive vestibular assessment could be completed at the initial visit and peripheral vestibular causes could be convincingly excluded based on clinical history, examination, and available testing. In contrast, children who were scheduled but did not attend follow-up visits may represent a heterogeneous group; in our healthcare setting, this is often interpreted as symptom resolution rather than disengagement from care, although objective confirmation of this assumption is not available. The absence of follow-up data in 32% of children highlights a system-level challenge in pediatric vestibular care. Structured recall protocols, predefined reassessment intervals for diagnoses that remain undefined, and improved parental counseling about the potential evolution of symptoms may improve longitudinal diagnostic accuracy. Importantly, extended follow-up proved critical for etiological clarification in a substantial proportion of patients, as diagnostic reclassification frequently occurred over time. This observation is consistent with previous reports, underscoring that delayed recognition of vestibular pathology is common in pediatric populations and supports the value of longitudinal reassessment [16].

### 4.3. Peripheral Etiologies

The increase in peripheral vestibular diagnoses observed after follow-up revision can be partly explained by the inherent challenges of pediatric vestibular assessment at the initial visit. Children do not always cooperate optimally, and comprehensive vestibular testing cannot consistently be completed during a single consultation, particularly in younger patients [17,18]. In addition, certain peripheral vestibular disorders such as benign paroxysmal positional vertigo (BPPV) may not be diagnosed at initial clinical presentation in pediatric patients, even when vestibular evaluation is performed, with diagnoses frequently established only after several weeks of symptom persistence and comprehensive follow-up evaluation, as shown in Brodsky’s work on pediatric concussion cases in which peripheral disorders were recognized on average 133 days after symptom onset rather than at first assessment [16]. On the other hand, pediatric BPPV may resolve rapidly, particularly in younger children, which can result in underdiagnosis if vestibular assessment is not performed promptly. A structured and timely vestibular evaluation pathway is therefore essential to ensure accurate identification and reliable epidemiological data.

### 4.4. Central Etiologies

In contrast to the baseline analysis, migraine-related vertigo emerged as the most frequent central diagnosis after revision. This evolution is clinically plausible, as migraine-associated vestibular symptoms in children may initially present without headache or fail to meet full diagnostic criteria. The observed evolution from initially unclassified presentations to migraine-related diagnoses supports the concept of pediatric vestibular migraine as a developmental disorder, in which the phenotypic expression may require longitudinal observation before diagnostic criteria are fully met. The introduction of the Bárány Society and International Headache Society consensus definitions for Vestibular Migraine of Childhood, probable Vestibular Migraine of Childhood, and Recurrent Vertigo of Childhood has provided a more robust framework for classification and reduced diagnostic ambiguity [19].

Recent comparative studies further demonstrate that these entities share overlapping yet distinguishable clinical profiles, supporting repeated clinical reassessment rather than early definitive labeling [10]. Our findings are consistent with this literature, indicating that migraine-related diagnoses are frequently revealed only through follow-up rather than at initial presentation.

### 4.5. Hemodynamic Etiologies

Hemodynamic mechanisms represent a clinically relevant and often under-recognized contributor to pediatric dizziness and vertigo. In our revised 2025 cohort, hemodynamic etiologies accounted for 16% (41/257), supporting the growing recognition that orthostatic intolerance syndromes and reflex syncope-related physiology can present predominantly as dizziness rather than frank syncope. This interpretation is consistent with contemporary pediatric autonomic literature, which describes orthostatic intolerance as common in children and adolescents, typically manifesting as dizziness, lightheadedness, fatigue, visual symptoms, and relief when supine [20]. Moreover, vasovagal syncope is widely reported as the most frequent cause of transient loss of consciousness in youth, accounting for the majority of pediatric syncope presentations—highlighting the high background prevalence of reflex/autonomic mechanisms in this age group [21]. In our cohort, tilt-table testing was performed in a small, clinically selected subgroup (13/257; 5%), and 62% (8/13) showed pathological results. While the sample is limited, this positivity rate is broadly in line with the high diagnostic yield reported in specialized pediatric syncope cohorts undergoing tilt testing, where positivity can exceed two-thirds depending on referral selection and protocol; for example, a recent pediatric syncope series reported a 75% positive tilt-table response among those tested [22]. Therefore, our tilt-table findings are most plausibly interpreted as reflecting targeted testing in patients with higher pre-test probability, rather than population-level prevalence. Clinically, these data support a structured approach in which hemodynamic symptoms (orthostatic dizziness, presyncope, palpitations, visual dimming, symptom relief with recumbency) prompt focused autonomic evaluation, whereas indiscriminate tilt testing in unselected dizziness populations may be less informative [20]. Future prospective studies should include systematic autonomic evaluation protocols to allow more precise subclassification of hemodynamic causes in pediatric vertigo.

### 4.6. Neuroimaging in Pediatric Vertigo

Neuroimaging is commonly obtained in children presenting with vertigo or dizziness, particularly when neurological signs, persistent headache, or trauma are present; however, its diagnostic yield in the absence of specific clinical indicators is modest. In a tertiary referral cohort of children imaged for vertigo, abnormalities were more likely to correlate with neurological deficits or persistent headaches, and routine imaging without such red flags provided limited additional diagnostic value [23].

Consistent with this literature, in our 2025 revised cohort, cranial CT was performed in 160 children (62% of the total cohort) and brain MRI in 146 children (57%). Although CT scans were frequently obtained, pathological CT findings were rare (1%), reflecting a low yield in an unselected dizziness population. MRI demonstrated a higher frequency of abnormalities (17%) likely due to its greater soft-tissue resolution and ability to detect subtle central pathologies when clinically indicated. These observations align with pediatric imaging data showing that CT contributes less to etiological diagnosis in vestibular presentations compared with MRI when neuroimaging is selectively applied based on clinical suspicion [23].

Importantly, improved etiological classification through follow-up may reduce reliance on early extensive diagnostic testing and support a more rational, stepwise diagnostic strategy.

### 4.7. Psychogenic Vertigo

Age-related differences play an important role in the etiological spectrum of pediatric vertigo. In our original 2021 cohort, psychogenic vertigo was observed predominantly in older children and adolescents, whereas younger children more often presented with peripheral or benign episodic vestibular disorders. This age-dependent distribution suggested that psychological and functional contributors to vertigo become increasingly relevant with advancing age, a pattern consistently reported in the literature.

This observation is supported by population-based data showing that vertigo in children aged 3–17 years is significantly associated with emotional, behavioral, and concentration difficulties, with stronger associations in adolescents compared with younger children [24]. Clinical studies focusing on somatoform and psychogenic vertigo similarly report that these diagnoses are rare in early childhood but emerge more frequently during school age and adolescence, often in the context of psychosocial stressors and recurrent or persistent symptoms [25].

In the present 2025 revision, psychogenic etiologies accounted for 10% (26/257) of cases, a proportion comparable to that reported in other tertiary pediatric cohorts. Although the revision did not aim to reanalyze age-stratified outcomes, the persistence of psychogenic diagnoses across follow-up is consistent with our 2021 finding that psychological contributors are more likely to be identified in older children, particularly when symptoms persist, and organic causes have been excluded. These findings underscore the importance of incorporating age-sensitive psychosocial assessment into the diagnostic pathway, especially during follow-up of adolescents with unexplained or recurrent vertigo.

### 4.8. Strengths and Limitations

#### 4.8.1. Strengths

The principal strength of this study is the longitudinal re-evaluation of the same pediatric cohort using prospectively collected clinical data, combined with a retrospective revision of etiological classification after extended follow-up. This design enabled a direct within-cohort comparison between baseline diagnoses and those established after prolonged clinical observation. The relatively large sample size and the multidisciplinary diagnostic approach, involving pediatric neurology, otolaryngology, radiology, and ophthalmology, further enhance the robustness of the findings. Additionally, the application of updated diagnostic concepts and contemporary classifications of pediatric vestibular disorders increases the clinical relevance and translational value of the revised analysis.

#### 4.8.2. Limitations

Several limitations should be considered when interpreting the results of this study. Although clinical data were collected prospectively, the etiological revision performed in 2025 represents a retrospective reassessment and therefore depends on the completeness and quality of available medical records. Not all children underwent follow-up evaluation: of the total cohort (N = 257), 52 children (20%) were not scheduled for follow-up based on clinical judgment at initial assessment, and 29 children (11%) did not attend the planned follow-up visit. Consequently, 32% of the cohort did not have a completed follow-up examination, which may have limited the ability to detect etiological changes in a subset of patients. In our cultural and healthcare context, non-attendance is often interpreted as reflecting symptom resolution and perceived clinical improvement; however, this assumption cannot be objectively verified, and alternative explanations cannot be excluded.

Furthermore, the duration of follow-up varied widely, ranging from 0 to 132 months, with a relatively short median. While extended follow-up enabled etiological reclassification in many children, shorter follow-up intervals in others may have reduced the likelihood of identifying diagnoses that emerge later in the clinical course.

Due to the absence of standardized follow-up intervals and systematically recorded time-to-reclassification data, we were unable to determine whether diagnostic refinement predominantly occurred within a specific early time window (e.g., within the first six months). Prospective studies incorporating structured reassessment timelines are needed to better define optimal follow-up intervals in pediatric vestibular disorders. The identification of baseline clinical predictors of diagnostic evolution would be of considerable clinical value. However, the present retrospective design did not allow formal predictive modeling. Future prospective cohort studies should aim to identify symptom patterns or clinical features associated with a higher likelihood of diagnostic reclassification.

Moreover, as this study was conducted at a single tertiary referral center, the findings may not be fully generalizable to primary or secondary care settings, where patient populations, referral patterns, and diagnostic resources may differ.

A further limitation concerns the absence of systematically recorded data on completion rates of specific vestibular tests and age-related differences in cooperation. Although repeat vestibular testing was performed in selected cases during follow-up and contributed to diagnostic refinement, detailed quantitative documentation of test repetition and age-dependent feasibility was not available for retrospective analysis. This may have influenced diagnostic classification in some patients.

Finally, the hemodynamic category included cases of orthostatic intolerance and reflex-mediated mechanisms; however, detailed subclassification (e.g., formal differentiation between POTS, orthostatic hypotension, and vasovagal mechanisms) was not systematically documented, limiting further granularity of analysis.

## 5. Conclusions

This study demonstrates that pediatric vertigo and dizziness are frequently evolving conditions, in which etiological clarification often requires longitudinal follow-up. Revision of data after extended follow-up markedly reduced the proportion of unclassified cases and led to a redistribution toward central, hemodynamic, psychogenic, and peripheral vestibular diagnoses. These findings highlight that diagnostic uncertainty at initial presentation should be anticipated and does not imply the absence of an underlying disorder.

Overall, a longitudinal, multidisciplinary approach is essential to improve diagnostic accuracy, reduce unnecessary investigations, and optimize clinical management in children presenting with vertigo and dizziness.

## Figures and Tables

**Table 1:** Diagnostic investigations and findings in the study cohort during the extended follow-up period. Percentages of normal and abnormal findings are calculated based on the number of children who underwent each specific diagnostic investigation.

Diagnostic Investigation	Children Examined, n (%)	Normal Findings, n (%)	Abnormal Findings, n (%)
Neurological examination	190 (74)	164 (86)	26 (14)
Electroencephalography (EEG)	94 (37)	85 (90)	9 (10)
Tilt-table test	13 (5)	5 (39)	8 (62)
Cranial computed tomography (CT)	160 (62)	158 (99)	2 (1)
Brain magnetic resonance imaging (MRI)	146 (57)	121 (83)	25 (17)
*Borrelia burgdorferi* serology	78 (30)	71 (91)	7 (9)
Ophthalmological examination	84 (33)	71 (85)	13 (15)

**Table 2:** Selected pediatric vertigo/dizziness studies reporting an unclassified/unknown etiology.

References	Year	Study Design/Setting	Sample (n)	Diagnostic Workup (Summary)	Unclassified/Unknown Etiology
Božanić Urbančič NB et al. Unraveling the Etiology of Pediatric Vertigo and Dizziness: A Tertiary Pediatric Center Experience. Medicina (Kaunas). PMID: 34064850 [8]	2021	Retrospective cohort; tertiary pediatric ENT center	257	Multidisciplinary evaluation; clinical exam ± audiovestibular testing and targeted ancillary investigations	43.6% (112/257) unclassified
Wiener-Vacher SR et al. Epidemiology of Vestibular Impairments in a Pediatric Population. Semin Hear. PMID: 30038452 [4]	2018	Retrospective clinical series; referral center for complete vestibular testing	1037 (balance disorders subgroup)	Complete vestibular test battery (canal + otolith function) adapted for pediatrics	Unknown etiology 19.6% (among etiologies of balance disorders with vestibular impairment)
Khater A et al. Unveiling the Diagnosis of Pediatric Dizziness in a Tertiary Care Setting. PMID: 40291364 [11]	2025	Retrospective cohort; tertiary care	40	Clinical evaluation with vestibular/neurological assessment and ancillary tests as indicated (e.g., EEG, MRI)	22.5% (9/40) diagnosis not ascertained
Fancello V et al. Vertigo and Dizziness in Children: An Update. Children (Basel). PMID: 34828738 [9]	2021	Systematic review (PRISMA) of recent pediatric vertigo studies	2470 (total across included studies)	Included heterogeneous diagnostic protocols across studies; summarizes etiologic distributions	Etiology unknown in 0.9% overall (as reported in review synthesis)
Davitt M; Delvecchio MT; Aronoff SC. The Differential Diagnosis of Vertigo in Children: A Systematic Review of 2726 Cases. Pediatr Emerg Care. 2020;36:368–371. doi:10.1097/PEC.0000000000001281 [1]	2020	Systematic review (2726 pediatric cases pooled)	2726	Across included studies, heterogeneous assessments (history/physical, bedside vestibular exam; variable audiology/imaging/lab/vestibular testing depending on source study)	Unidentified/idiopathic: 11.7% (credible interval reported in review).
Lorente-Piera J, et al. Clinical Profile, Trends, and Management in Pediatric Patients with Audiovestibular Disorders. Audiol Res. 2024. PMID: 39194415 [12]	2024	Retrospective cohort; pediatric ENT/audiovestibular setting	117	Audiovestibular evaluation; vHIT/VEMP in subset; imaging performed in 64.1% (diagnostic in 34.7%)	Idiopathic (no identified cause): 7.69% (*n* = 9).
Rey-Berenguel M, et al. Diagnostic Yield of the New Bárány Society Criteria for Pediatric Episodic Vestibular Syndrome. J Clin Med. 2025 [13]	2025	Observational cross-sectional; tertiary pediatric vertigo clinic; EVS focus	109 (EVS cohort)	Standardized neuro-otological exam; audiovestibular tests and imaging as needed; classification using ICHD-3 vs. Bárány 2021 criteria	Undetermined EVS without hearing loss reduced from 35.78% to 16.51% after applying Bárány 2021 criteria.

## Data Availability

The data supporting the findings of this study are stored in a secure local database and are available from the corresponding author upon reasonable request. Access to the data is restricted due to patient privacy and ethical considerations.

## Abbreviations

The following abbreviations are used in this manuscript:

EEG	Electroencephalography
CT	Computerised tomography
MRI	Magnetic resonance imaging
BPPV	Benign paroxysmal positional vertigo
BPV	Benigne pediatric vertigo
